# Acute Postnatal Inflammation Alters Adult Microglial Responses to LPS that Are Sex-, Region- and Timing of Postnatal Inflammation-Dependent

**DOI:** 10.21203/rs.3.rs-4565866/v1

**Published:** 2024-06-27

**Authors:** Maria Nikodemova, Jose R Oberto, Mackenzie R Berschel, Alysha L Michaelson, Jyoti J Watters, Gordon S Mitchell

**Affiliations:** University of Florida; University of Florida; University of Florida; University of Florida; University of Wisconsin; University of Florida

## Abstract

**Background.:**

Adverse events in early life can have impact lasting into adulthood. We investigated the long-term effects of systemic inflammation during postnatal development on adult microglial responses to LPS in two CNS regions (cortex, cervical spinal cord) in male and female rats.

**Methods.:**

Inflammation was induced in Sprague-Dawley rats by lipopolysaccharide (LPS, 1 mg/kg) administered intraperitoneally during postnatal development at P7, P12 or P18. As adults (12 weeks of age), the rats received a second LPS dose (1 mg/kg). Control rats received saline. Microglia were isolated 3 hours post-LPS from the cortex and cervical spinal cord. Gene expression was assessed via qRT-PCR for pro-inflammatory (IL-6, iNOS, Ptgs2, C/EBPb, CD14, CXCL10), anti-inflammatory (CD68, Arg-1), and homeostatic genes (P2Y12, Tmemm119). CSF-1 and CX3CL1 mRNA was analyzed in microglia-free homogenates.

**Results.:**

Basal gene expression in adult microglia was largely unaffected by early life LPS. Changes in adult microglial pro-inflammatory genes in response to LPS were either unchanged or attenuated in rats exposed to LPS during postnatal development. Ptgs2, C/EBPb, CXCL10 and Arg-1 were the genes most affected, with expression levels significantly downregulated vs control rats without postnatal LPS exposure. Cortical microglia were affected more by postnatal inflammation than spinal microglia, and males were more impacted than females. Overall, inflammatory challenge at P18 had the greatest effect on adult microglial gene expression, whereas challenge at P7 had less impact. Microglial homeostatic genes were unaffected by postnatal LPS.

**Conclusions.:**

Long-lasting effects of postnatal inflammation on adult microglia depend on the timing of postnatal inflammation, CNS region and sex.

## Introduction

Early life adverse events are associated with long-lasting health effects, including increased risk for neurodevelopmental, behavioral, and adult-onset neurodegenerative disorders (1–3). Both humans and rodents are born with immature central nervous and immune systems that continue developing during the early postnatal period. Thus, infections or stress experienced during this critical developmental window may interfere with normal development, reprogramming the immune response to later challenges as adults (4–6). For example, prenatal and neonatal infections are linked to schizophrenia, autism, ADHD and other behavioral disorders (3, 7). Postnatal inflammation induced by lipopolysaccharide (LPS) increases seizure risk in adult rats (8). Prenatal and neonatal inflammation are also associated with breathing deficits, such as disruption of normal respiratory control mechanisms, such as neuroplasticity (9–11).

Postnatal CNS development is characterized by synaptogenesis, synaptic pruning, neuronal apoptosis and myelination, with microglial cells playing a critical role in these processes (12–14). During this period, microglia undergo morphological and phenotypic transformation, reflecting their developmental trajectory and differing roles at different developmental stages. Microglia have a high proliferation rate during the first 2 postnatal weeks, when their density is twice that found in the adult CNS (15, 16). Microglial numbers start declining in the third postnatal week, reaching adult numbers by week 6 (15). Certain inflammatory mediators, such as TNFa and iNOS, are elevated in P3 microglia, whereas P21 microglia express higher CD11b, TLR4 and FcRg levels (17). In the healthy adult CNS, microglia maintain neural homeostasis and immune function (18, 19). On the other hand, they are also involved in most CNS pathologies and/or traumas, exhibiting both beneficial and detrimental activities. Thus, it is important to understand how their activities are shaped by prior experiences, particularly in early postnatal life.

In this study we investigated long-lasting effects of mild acute systemic inflammation induced by intraperitoneal administration of LPS at different times during postnatal development (P7, P12, P18) on microglial responses to the same challenge in adulthood. We further evaluated differences in CNS regional susceptibility to postnatal inflammation, comparing microglia in the cortex versus cervical spinal cord. Spinal cord microglia are seldom considered in studies of early life challenges and adult function despite the fact that the spinal cord is a key region engaged in the neural control of breathing (9, 11). Lastly, we determined if LPS at different times after birth differentially affects adult microglia, and if these differences are sex dependent.

## Methods

### Animals

All experiments were approved by the University of Florida Institutional Animal Care and Use Committee. Pregnant Sprague-Dawley rats were purchased from Envigo (IN, USA). Rats were housed at AAALC-accredited animal facility under standard conditions with a 12-hour light/dark cycle and *ad libitum* food and water.

### Experimental design

**Figure 1:**
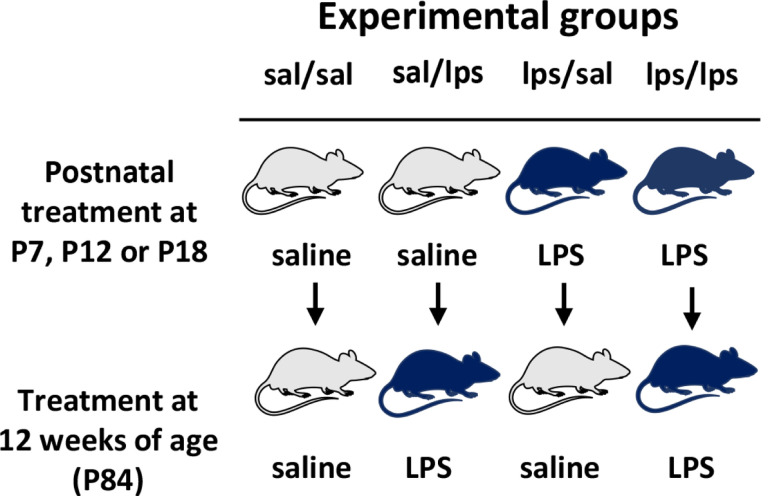
Experimental design. Pups in each litter were randomly divided into two groups receiving either i.p. saline or LPS (1mg/kg) at P7, P12 or P18. At 12 weeks of age (P84), animals received either saline or LPS (1 mg/kg). Three hours post-injection animals were perfused followed by spleen, cortex and cervical spinal cord harvest. Fresh CNS tissue were used for microglial isolation and gene expression analysis by qRT-PCR. The experimental group name depicts postnatal/P84 treatment.

The experimental design and groups are depicted in Fig. 1. At the age of P7, P12 or P18, pups in each litter were randomly assigned to a group receiving either saline or lipopolysaccharide (LPS) at the dose of 1 mg/kg administered by i.p. injection. Each experimental group included animals from 2–3 litters to account for inter-litter differences. Animals were grown up to 12 weeks of age when they received a second challenge with either saline or LPS (1mg/kg). LPS was administered between 9–10am in postnatal rats and 8.30am −12pm in adult rats to avoid potential circadian effects on inflammatory responses. We did not observe any behavioral or body changes (posture, swelling) after LPS administration in either age. Table 1 depicts the number of rats in each experimental group. Adult animals were deeply anesthetized by isoflurane inhalation 3 hours after saline or LPS administration, and intracardially perfused with ice-cold PBS followed by spleen, cortex and cervical spinal cord dissection. CNS tissues were immediately used for microglia isolation. Spleen tissues were frozen and stored at −80°C until further use.

### Microglia isolation

Microglia were isolated from fresh tissues using the immunomagnetic cell separation method described in detail previously (20). All reagents were obtained from Miltenyi Biotech, Germany. Tissues were mechanically and enzymatically dissociated into single cell suspensions using a papain Neural Tissue Dissociation Kit. After myelin removal by centrifugation in Debris Removal Solution, cells were incubated with CD11b magnetic beads followed by separation in a magnetic field using MS columns. Both CD11b^+^ (microglia) and CD11b^−^ (non-microglia) fractions were collected. This method consistently yields > 96% microglial cell purity (20).

### RNA extraction and qRT- PCR

Total RNA was extracted from cells using TRIZOL reagent (Invitrogen) following the manufacturer’s protocol. Trans-sectional spleen slices were ultrasonically homogenized in 1 ml TRIZOL reagent followed by total RNA extraction. Extracted RNA (250 ng) was used for cDNA synthesis using iScript Advanced cDNA Synthesis kit (BioRad). All primers for quantitative PCR were obtained from BioRad (Table 2) together with SsoAdvanced Universal Real-Time PCR Supermix. After normalization to housekeeping gene hprt1, gene expression was analyzed using the ddCt method.

### Statistical analysis

Data were analyzed using SigmaPlot (descriptive statistics, ANOVA) and SAS (regression analysis) software. Data are expressed as mean ± standard error of mean (SEM). Group differences were considered significant if p < 0.05. Significance scores are * for p < 0.05, ** for p < 0.01, *** for p < 0.001, no symbol for not significant.

The “dissimilarity” in adult LPS responses between animals with and without prior LPS exposure was analyzed by Bray-Curtis (BC) dissimilarity index, a statistical tool quantifying the dissimilarity in species composition between two different sites commonly used in ecological and microbiological studies. In this instance we used experimental groups as “sites” and mRNA expression as “species”. The BC dissimilarity index was calculated as follows:

$$\text{BCij}=1-\frac{2Cij}{Si+Sj}$$

Where i,j are experimental groups; Cij is the sum of the lesser count of each species (mRNA); Si and Sj are the total number of specimen in group. The BC dissimilarity index is bounded between 0 and 1, where 0 mean two groups are identical, and 1 means two groups are completely different.

## Results

### Early-life immune challenge does not affect adult body weight

**Figure 2:**
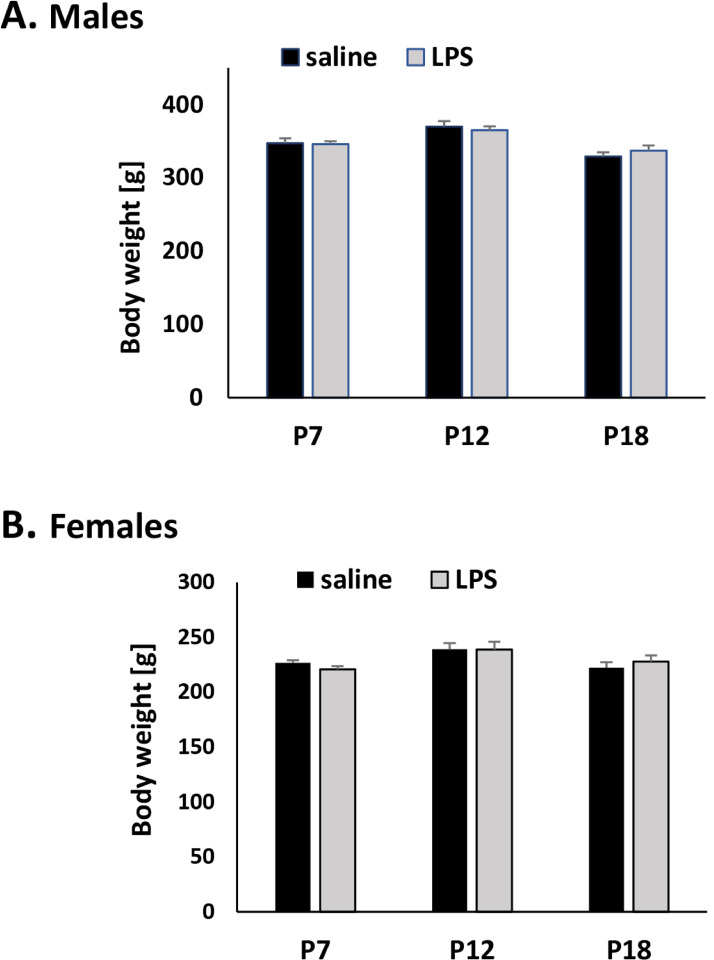
The effect of postnatal LPS challenge on adult body weight. Body weight was recorded at P84 before adult challenge with saline or LPS. The legend (saline, LPS) indicates postnatal treatment. We did not observe any significant changes in body weight in males (A) or females (B) receiving LPS postnatally compared to rats receiving only saline.

Inflammation induced by LPS during postnatal development did not affect body weight (BW) at 12 weeks of age (Fig. 2). Body weights were recorded before the second LPS or saline challenge. The average BWs of males receiving saline or LPS during postnatal development (P7, P12, P18) were 349 g and 350 g, whereas the average BWs of females were 229 g and 229 g, respectively.

### Adult microglial responses to LPS differ by CNS region and sex

**Figure 3:**
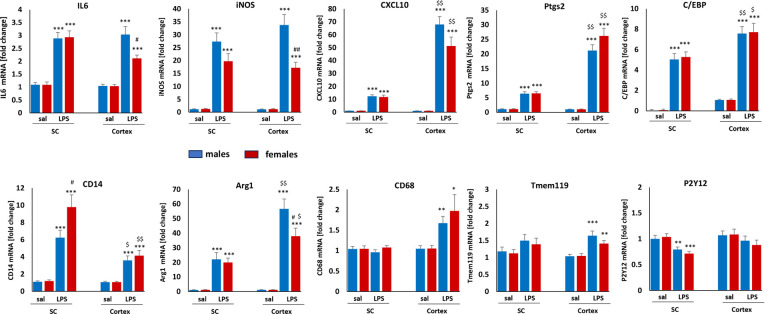
Adult microglial responses to LPS. Rats receiving only saline during postnatal development were used as controls to determine adult microglia response to LPS (1mg/kg) in cortex or cervical spinal cord. Gene expression was analyzed 3 hours post LPS administration. Statistical Significance is illustrated as follows: * LPS vs saline; # males vs females; $ cortex vs spinal cord. One symbol=p<0.05, two symbols p<0.01, three symbols p<0.001

We evaluated adult microglial responses to systemic LPS in adult rats that had received saline during postnatal development (controls) 3 hours post-LPS administration, including genes associated with inflammation, phagocytosis and microglial homeostasis (Table 2). Three hours post-LPS treatment, IL6, iNOS, CXCL10, Ptgs2, C/EBP, CD14 and Arg1 were significantly upregulated in adult male and female microglia in both cortex, cervical spinal cord, although the magnitudes of individual gene responses differed (Fig. 3). Cortical microglia from both sexes had significantly higher levels of CXCL10, Ptgs2, C/EBPb and Arg1 after LPS *versus* spinal microglia; the CD14 response was significantly lower in cortical microglia. Increased CD68 and Tmem119 expression post-LPS was detected only in cortical microglia. On the other hand, P2Y12 was downregulated after LPS exclusively in spinal microglia. Cortical microglia also exhibited larger, sex-dependent differences than spinal microglia; male microglia had significantly higher responses in IL6, iNOS and Arg1 after LPS versus females. In spinal microglia, the only sex difference identified was in CD14, which was higher in female versus male microglia. We did not detect changes in TNFa, CCL2 or IL1b mRNA 3 hours post-LPS (data not shown).

### Early-life inflammation has little effect on basal gene expression in adult microglia

To determine if postnatal inflammation had long-lasting effects on adult microglia, we evaluated basal gene expression in 12-week-old rats exposed to LPS at P7, P12 or P18. In microglia from cortex and spinal cord, a single LPS challenge during postnatal development had minimal effects on basal expression of any gene evaluated here (Table 3). Spinal microglial IL6 expression was downregulated in females exposed to LPS at P7, and in males exposed to LPS at P18. In cortical microglia, there were no significant changes in gene expression in females; in male cortical microglia, CXCL10 mRNA was elevated in rats receiving LPS at P7, and CD14 was downregulated in adult rats exposed to LPS at P18.

#### LPS-evoked gene expression differs with early-life exposure timing, CNS region and sex.

**Figure 4:**
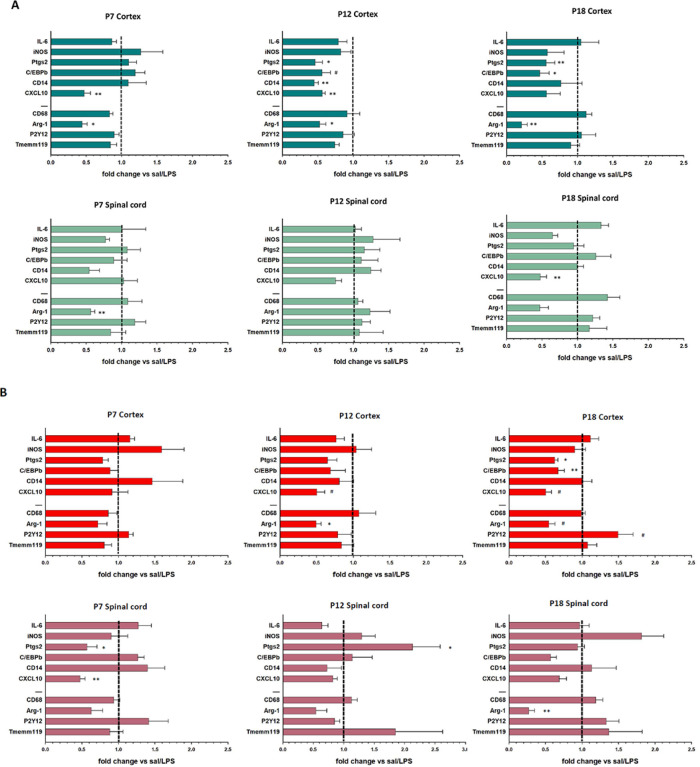
The effect of postnatal inflammation on adult microglial responses to LPS. Animals received first dose of LPS (1 mg/kg) either at P7, P12 or P18. Second LPS (1mg/kg) was administered at P84 (12 weeks of age). Males (**A**) and females (**B**)microglial responses in cortex and cervical spinal cord were compared to control animal receiving LPS only at P84 (set to 1, middle dashed line). Genes are grouped as pro-inflammatory (IL-6, iNOS, Ptgs2, C/EBPb, CD14, CXCL10), anti-inflammatory and homeostatic (CD68, Arg-1, P2Y12, Tmemm119). P-value indicates significant difference in LPS response between control and experimental animals. *p<0.05, **p<0.01, ***p<0.001; # indicate p-value >0.05 but <0.085

**Figure 5:**
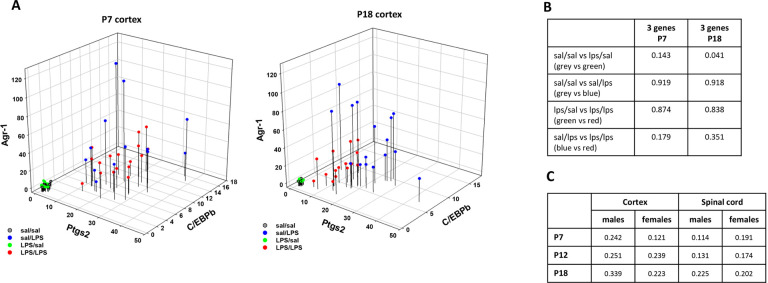
Bray-Curtis analysis of differences in microglial responsiveness. (**A**) Illustration of C/EBPb, Arg-1 and Ptgs2 mRNA expression in cortical microglia. To visualize the impact of postnatal LPS challenge on adult microglial responses to second LPS challenge, the expression of 3 most affected genes is compared in microglia exposed to LPS or saline at P7 (left) and P18 (right). The basal expression of these genes was comparable in adult animals receiving saline (grey) or LPS (green) during postnatal treatment. LPS at 12 weeks of age increased expression of all three genes (red, blue), however, we do not see a clear separation between animals receiving saline (blue) or LPS (red) at P7. On the other hand, there is a distinct grouping based on postnatal treatment received at P18 suggesting differential response of microglia with and without prior LPS exposure. Data from both sexes are pooled. (**B**)Sensitivity analysis of quantifying group differences by Bray-Curtis dissimilarity index. As expected, the dissimilarity index was highest comparing basal and LPS induced gene expression. On the others hand, the index was low (suggesting greater similarity) for basal gene expression in animals receiving saline or LPS postnatally. (**C**) Bray Curtis dissimilarity index including all 10 genes comparing adult responses to LPS between control animals (postnatal saline) or animal receiving LPS at P7, P12 or P18. The 3D figures help to visualize different dissimilarity indexes (for example 0.919 vs 0.143).

The response of the homeostatic microglial genes P2Y12 and Tmem119 to LPS as adults was unaffected by postnatal LPS exposure in either CNS region (Figs. 4 & 5). Similarly, LPS responses in CD68 (anti-inflammatory gene associated with phagocytosis) were unaffected by postnatal LPS history, except in male cortical microglia which was significantly attenuated by LPS exposure at P7 (Fig. 4A). LPS in naïve adult rats without developmental LPS exposure significantly increased Arg-1 expression (anti-inflammatory gene) in male and female microglia from both CNS regions (Fig. 3). Developmental LPS, regardless of postnatal timing, significantly blunted this response in both cortex and spinal cord by up to 80%, although this decrease did not always reach statistical Significance in females (Fig. 4B). The only exception observed was in spinal microglia from males exposed to LPS at P12, where the Arg-1 response was not altered.

The effects of postnatal LPS on adult microglial inflammatory responses were dependent on CNS region, sex and time of postnatal LPS exposure. LPS-evoked inflammatory gene expression was attenuated in cortical microglia in males that had been exposed to LPS at P12 and P18 versus controls (i.e. saline at P12 or P18) (Fig. 4A). The most affected genes were CXCL10, C/EBPb and Ptgs2. P7 LPS treatment had lesser effects on male adult microglia where only the CXCL10 response was attenuated significantly. In contrast, spinal microglial inflammatory responses were largely unaffected by postnatal LPS in males. In females, cortical microglial inflammatory responses were largely unaffected by postnatal LPS at P7 or P12, while expression of several pro-inflammatory genes were attenuated by LPS at P18 (Fig. 4B). Female spinal microglia were most affected by P7 LPS exposure. However, larger variability in gene expression was observed in females versus males.

To quantify similarity (or dissimilarity) of adult microglial responsiveness to LPS with and without postnatal LPS challenge, we calculated the Bray-Curtis dissimilarity (BC) index that reflects differences in expression of all 10 genes studied. The advantage of using this index is that it encompasses all differences in gene expression even if individual changes are not statistically significant, which is important since an accumulation of small differences across multiple genes could lead to biologically meaningful effects. Because this statistic is typically used in ecological studies, we first performed sensitivity analyses to determine if this statistic was suitable to reflect dissimilarity between microglial gene expression among groups. We performed a sensitivity analysis using the expression of 3 genes (C/EBPb, Ptgs2 and Arg-1), comparing cortical microglia from rats receiving LPS at P7 or P18 versus control rats receiving saline. As illustrated in the 3D graphs in Fig. 5A, basal gene expression was comparable, regardless of postnatal exposure (sal/sal and LPS/sal groups), and adult LPS challenge upregulated all 3 genes in sal/LPS and LPS/LPS groups. Whereas P7 LPS had little effect on LPS-induced gene responses in adults, P18 LPS challenge attenuated adult microglial responses. Figure 5 illustrates that gene expression in sal/LPS and LPS/LPS treatment groups were more similar when the postnatal LPS challenge occurred at P7 and were more distinct when the LPS challenge occurred at P18, indicating greater dissimilarity. The Bray-Curtis dissimilarity indexes comparing basal gene expression in control animals (sal/sal) versus rats exposed to LPS at P7 or P18 (LPS/sal groups) were 0.143 and 0.041, indicating high similarity between groups (Fig. 5B). In contrast, the indexes were 0.919 and 0.918 (for sal/sal & sal/LPS) for rats that had received saline at P7 vs P18 in response to LPS as adults; and 0.874 and 0.834 (for LPS/sal vs LPS/LPS) for animals receiving LPS at P7 or P18, indicating high dissimilarity at 12 weeks between groups receiving postnatal saline or LPS (3D figure in Fig. 5A). The Bray-Curtis index comparing adult LPS responses in rats receiving postnatal saline or LPS (sal/LPS vs LPS/LPS) was 0.179 for P7 and 0.351 for P18, suggesting that the dissimilarity in LPS response was higher if postnatal LPS exposure occurred at P18. The 3D figures also help visualize different Bray Curtis dissimilarity indexes. Altogether, this sensitivity analysis suggests that the Bray Curtis dissimilarity index is suitable for analyzing similarities/dissimilarities in gene expression between microglia from rats exposed to different conditions.

To compare how the adult LPS response was affected by prior postnatal LPS exposure, using all 10 genes, we calculated the Bray Curtis dissimilarity index between rats receiving postnatal saline (sal/LPS) or LPS at P7, P12 or P18 (LPS/LPS). The highest dissimilarity index (0.339) was found for male cortical microglia with P18 LPS (Fig. 5C), suggesting that the adult LPS response was 33.9% dissimilar (or 76.1% similar) to the LPS response in microglia from adult males without postnatal LPS challenge. In the cortex, male microglia were more affected by postnatal LPS than females, as they had a higher dissimilarity index regardless of postnatal LPS timing.

Adult male spinal microglial gene expression was only nominally altered by P7 or P12 LPS exposure and was less affected by LPS at P18 versus cortical microglia. Spinal microglial gene expression in females was less affected by LPS challenge at P12 and P18 versus cortical microglia, but more affected by P7 LPS. Overall, the largest long-term effect of postnatal inflammation occurred after P18 LPS exposure in both CNS regions.

### Attenuated LPS-induced CSF-1 gene expression in non-microglial cell fraction

**Figure 6:**
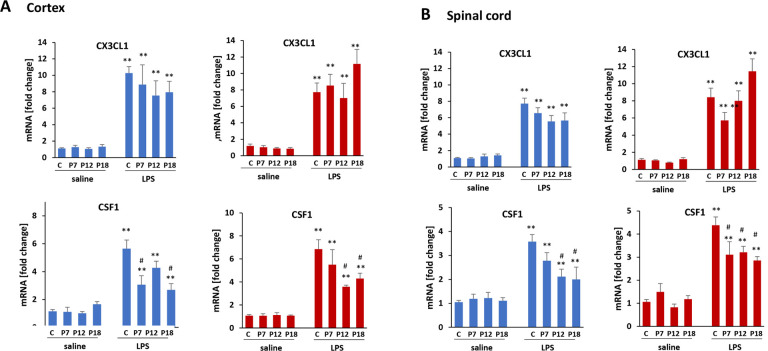
The effect of postnatal inflammation on CX3CL1 and CSF1. The expression of CX3CL1 and CSF1 was assessed in microglial-free tissue homogenates in cortex (**A**) and cervical spinal cord (**B**). There was no difference in basal expression of these genes (saline) in adult rat with and without postnatal LPS exposure. P7, P12, P18 indicates when LPS was administered. “C” indicates that animals received only saline postnatally. LPS at 12 weeks of age induced significant upregulation on CX3Cl1 and CSF1 in males (**blue**) and females (**red**) in both CNS regions. CX3CL1 and CSF1 were significantly upregulated by LPS both in cortex and spinal cord in animals receiving saline (C) or LPS (P7, P12, P18) postnatally. **p<0.01 vs saline; ^#^p<0.05 vs LPS in “C” animals

In the CD11b negative cell fractions (neurons, astrocytes, oligodendrocytes, endothelial cells, and others) from cortex and cervical spinal cord, we analyzed expression of CX3CL1 (fractalkine) and CSF-1 (colony stimulating factor 1), important regulators of microglial activities (Fig. 6). CX3CL1 is expressed primarily by neurons whereas CSF-1 is produced by neurons and astrocytes. LPS significantly upregulated both genes in adult cortical and spinal CD11b negative cell fractions; the timing of postnatal LPS challenge had no effect on basal or LPS-evoked CX3CL1 expression. On the other hand, the LPS-evoked increase in adult CSF-1 expression in cortical and spinal CD11b negative cell fractions was attenuated by postnatal LPS, although this effect did not reach statistical Significance in cortex from males treated with LPS at P12 (p = 0.243) or females treated with LPS at P7 (p = 0.389), nor in spinal fractions from males treated with LPS at P7 (p = 0.169).

### Inflammation at P18 significantly affected adult spleen response to LPS

**Figure 7:**
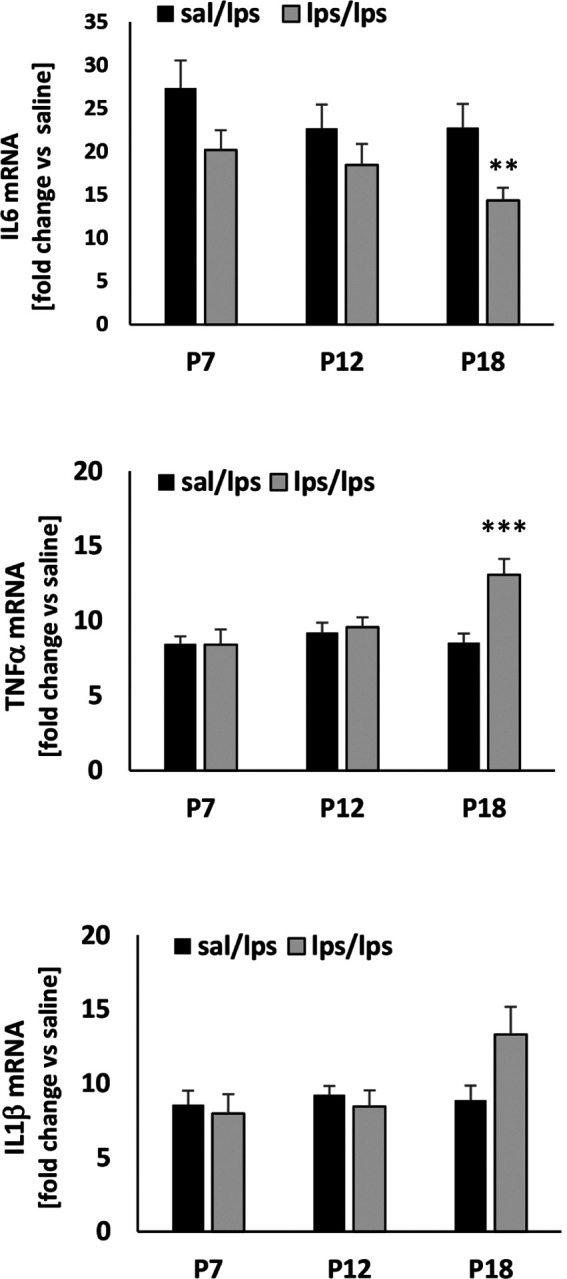
The effect of postnatal inflammation on adult spleen responses to LPS. Animals were exposed LPS or saline at P7, P12 or P18. IL6, TNFa and IL1b mRNA was analyzed in spleens in response to LPS at 12 weeks of age. LPS induced significant upregulation of inflammatory cytokines in all treatment group. **p<0.01, ***p<0.001 vs sal/LPS

Because neuroinflammation was induced by systemic inflammation induced via intraperitoneal LPS injections, we evaluated whether the peripheral immune response to LPS in adults was affected by postnatal LPS challenge. We did not observe any differences in gene expression for IL6, TNFa or IL1b in basal (not shown) or LPS-evoked response of spleens from rats with P7 or P12 LPS challenge (Fig. 7). However, in adult spleens from animals exposed to LPS at P18, LPS-evoked IL6 mRNA levels were attenuated whereas TNFa mRNA levels were exaggerated. We observed an apparent increase in LPS-evoked IL1b gene expression in spleens from rats treated with LPS at P18, but this change did not reach statistical Significance (p = 0.136).

## Discussion

Microglia contribute to many important biological functions in the developing and adult CNS including neurogenesis, myelination, synaptic remodeling, synaptic pruning and synaptic plasticity (14, 19, 21). Microglial reactivity is observed with CNS pathology or injury, exerting both harmful and beneficial effects depending on the microenvironment and specific pathology (19, 22). The immune system, including microglia, is immature post-birth and continues to develop for up to 7 weeks (23). While development is largely guided by genetics, adverse events in early life can significantly impact development, altering immune responsiveness later in life (23, 24). Since development of the CNS immune system progresses throughout the postnatal period, the same adverse event (e.g., acute inflammation) experienced at different developmental stages may differentially affect development and permanently affect adult microglia.

In healthy adult rats, microglia from the cortex and cervical spinal cord exhibit multiple differences, including differential sensitivities to systemic inflammation. For example, microglial density in the cortex is considerably higher than in the cervical spinal cord (25). We now report that LPS-evoked upregulation of CXCL10, Ptgs2 and Arg1 is more robust in cortical versus spinal microglia. C/EBPb, a transcription factor regulating expression of key pro-inflammatory genes (26, 27), is also more elevated in cortical versus spinal microglia. We detected few sex-dependent differences in response to LPS, but females had a weaker IL6 and iNOS gene expression response in cortical microglia, confirming that inflammation in males may be more robust (28). While we did not observe major differences in basal microglial gene expression in the cortex or spinal cord from adult rats that had received postnatal LPS, their responses to a second LPS challenge at 12 weeks of age were impacted significantly. Thus, whereas adult microglia may appear unaffected by a history of LPS challenge during basal conditions, long-lasting effects of a prior inflammatory insult can be unmasked by a new LPS challenge to the immune system. Thus, microglial reactivity to adult-onset pathology or trauma may differ between animals with and without a history of developmental immune challenge. However, new studies are needed to fully understand the impact of early life events on adult-onset disease susceptibility, severity or progression.

We found sex- and region- specific vulnerability to early life inflammatory challenge. For example, cortical microglia were more affected in males regardless of the timing of postnatal challenge. Spinal microglia were more affected in females, but only with P7 or P12 LPS challenge.

The postnatal timing of the inflammatory insult appears to be important in terms of the magnitude of gene expression responses to adult LPS challenge, and the specific genes affected. In cortex, an attenuated inflammatory response of adult microglia was generally observed in both in females and males that had P12 or P18 LPS challenge (P7 LPS had lesser effects). Spinal microglia were less affected by postnatal LPS and, in contrast to cortex, females were more affected than males. While several differences in response to LPS between control (no prior LPS) *versus* rats with post-natal LPS challenge did not reach statistical Significance, it remains possible that an accumulation of smaller, difficult to detect changes across multiple inflammatory genes can still have significant biological effects. With this rationale, the Bray-Curtis dissimilarity index suggests that LPS challenge at P18 has the largest long-term effect on adult microglial responsiveness to a second LPS challenge in adulthood.

Microglia from females tended to have larger variability in LPS response *versus* males, potentially explaining why some apparent changes in females did not reach statistical Significance. Estrogens have anti-inflammatory activities and can modify microglial responses to LPS (29, 30). Because we did not evaluate the estrous cycle in our females at the time of adult LPS challenge, different estrous cycle phases within experimental groups could contribute to variability in LPS responsiveness. However, it is less likely that this factor can account for differences between cortical and spinal microglia in females, unless there are regional differences in sensitivity to sex hormones; such effects have not been reported to our knowledge. It is important to note that litter-dependent variability in LPS response was apparent, potentially contributing additional variability in gene expression; however, we lack sufficient statistical power to analyze this effect.

Research on maternal or early life inflammation predominantly focuses on long-term effects in the brain; literature studying consequences for the spinal cord is sparce (31, 32). Our data show that inflammation in early postnatal life affects cortical and spinal microglia differently. Similar differences are possible in other CNS regions.

Several mechanisms of microglial reprogramming induced by postnatal inflammation have been suggested, including HPA axis dysfunction and epigenetic modifications (2–6). Although we did not investigate possible mechanisms of modified microglial responses to LPS in this study, we did analyze CSF-1 and CX3CL1, important regulators of microglial survival and activities (33–35). CX3CL1 exerts anti-inflammatory effects, although high concentrations of this cytokine can elicit pro-inflammatory responses (35). LPS significantly upregulated mRNAs for both molecules. While CX3CL1 gene expression was unaffected by prior LPS exposure, CSF-1 expression was attenuated in both CNS regions and both sexes. CSF-1 is necessary for microglial proliferation and survival, and pharmacological blockade of the CSF-1 receptor almost completely depletes microglia (36, 37). Thus, it is possible that weaker CSF-1 responses in rats challenged with LPS during postnatal development may limit adult microglial inflammatory responses. Multiple mechanisms likely contribute to altered adult microglial LPS responsiveness, including actions within microglia *per se* as well as effects on nearby cells, such as neurons and astrocytes. Both neurons and astrocytes interact with microglia; thus, changes in neuronal or astrocytic function could indirectly regulate microglial activities in rats with a history of developmental inflammation. Because LPS does not cross the blood-brain barrier, neuroinflammation induced by systemic LPS is largely mediated via peripheral inflammation. Since previous encounters with specific antigens induce immune tolerance, it is possible that a postnatal LPS alters peripheral inflammatory responses in adult rats, indirectly regulating adult microglial responses. Although we analyzed only select genes in the spleen, the peripheral immune response did not appear to be affected in rats with LPS exposure at P7 or P12, whereas responses for LPS at P18 were mixed. Thus, we cannot exclude the possibility that at least some of the differences reported here reflect changes in peripheral inflammatory responses.

### Conclusions:

Acute inflammation induced by LPS during postnatal development has long-lasting effects on adult microglial responses to subsequent LPS challenge. These effects are gene specific and depend on sex, CNS region and the timing of the postnatal LPS challenge. This study raises multiple questions for future research. It will be important to determine: 1) what other genes are impacted by postnatal inflammation; 2) how long postnatal inflammation effects persist; 3) whether postnatal inflammation alters other adult cells; 4) whether postnatal inflammation alters responses to other challenges as adults (e.g., hypoxia); and 5) the consequences of altered adult microglial responses for adult-onset neurodegenerative disorders or acquired neural injuries.

## Data Availability

The data presented in this study are available from the corresponding author upon reasonable request.
